# Long-Term Safety and Survival Outcomes of [^225^Ac]Ac-PSMA (Prostate-Specific Membrane Antigen) and [^225^Ac]Ac-/[^177^Lu]Lu-PSMA (TANDEM) Radioligand Therapy (PRLT) in Metastatic Castration-Resistant Prostate Cancer

**DOI:** 10.3390/cancers17030405

**Published:** 2025-01-26

**Authors:** Elisabetta Perrone, Alessandro Giordano, Maria Lucia Calcagni, Lucia Leccisotti, Roberto Moretti, Aleksandr Eismant, Kriti Ghai, Tanay Parkar, Aditi Mishra, Axel Heidenreich, Ralph M. Wirtz, Jörg Müller, Lukas Greifenstein, Richard P. Baum

**Affiliations:** 1 CURANOSTICUM Wiesbaden-Frankfurt, Center for Advanced Radiomolecular Precision Oncology, 65191 Wiesbaden, Germany; eismant@curanosticum.de (A.E.); ghai@curanosticum.de (K.G.); parkar@curanosticum.de (T.P.); aditi.mishra@gmail.com (A.M.); greifenstein@curanosticum.de (L.G.); baumrp@gmail.com (R.P.B.); 2 Institute of Nuclear Medicine, Università Cattolica del Sacro Cuore, 00168 Rome, Italy; alessandro.giordano@unicatt.it (A.G.); marialucia.calcagni@policlinicogemelli.it (M.L.C.); lucia.leccisotti@policlinicogemelli.it (L.L.); 3 Unit of Nuclear Medicine, Department of Diagnostic Imaging, Radiation Oncology and Hematology, Fondazione Policlinico Universitario Agostino Gemelli IRCCS, 00168 Rome, Italy; 4 Unità della Fisica per le Scienze della Vita, Fondazione Policlinico Universitario Agostino Gemelli IRCCS, 00168 Rome, Italy; robertomoretti1993@gmail.com; 5 Department of Urology, Uro-Oncology, Robot-Assisted and Reconstructive Surgery, Faculty of Medicine, University of Cologne, University Hospital Cologne, 50937 Cologne, Germany; axel.heidenreich@uk-koeln.de; 6 Institute of Pathology, St. Elisabeth Hospital Köln-Hohenlind, 50935 Cologne, Germany; ralph.wirtz@stratifyer.de; 7 Department of Neurology, Vivantes Klinikum Spandau, Charité–Universitätsmedizin, 10117 Berlin, Germany; j.mueller@berlin.de

**Keywords:** prostate cancer, PSMA, PSMA Radioligand Therapy (PRLT), Actinium-225, TANDEM PRLT, safety, survival

## Abstract

This study aims to retrospectively assess the safety of [^225^Ac]Ac-PSMA-PRLT, both as monotherapy and in combination (TANDEM) with Lutetium-177, concerning tolerance after the radiopharmaceutical administration and long-term safety, its impact on salivary glands’ function, overall survival (OS), and follow-up duration. Between December 2020 and September 2024, 89 patients received a total of 151 cycles of [^225^Ac]Ac-PSMA-PRLT. Patients with at least one follow-up (n = 71) were included in the analysis to evaluate xerostomia, as well as long-term hematological, renal, and hepatic toxicities, graded according to CTCAE v5.0. The most common adverse event after the radiopharmaceutical administration was flare pain (n = 16). As of the time of analysis, 68 patients had passed away (76.4%; range of survival 5 days to 39 months, median 7 months), while 21 patients were still alive (23.6%; follow-up duration: 1–33 months). Severe (G3/G4) long-term adverse events were rare, with 15 cases of G3 anemia (21.1%), 6 cases of G3 leukocytopenia (8.4%), and 14 cases of G3/G4 thrombocytopenia (19.7%). Hematological toxicity was primarily associated with severe bone marrow involvement or prior chemotherapy. Additionally, one case of G3 nephrotoxicity (1.4%) and six cases of G3 hepatotoxicity (8.4%) were observed. Only nine patients (12.7%) reported de novo xerostomia (G1/G2). In conclusion, this study demonstrates that [^225^Ac]Ac-PSMA PRLT, both as monotherapy and combined with [^177^Lu]Lu-PSMA as TANDEM PRLT, is generally safe in terms of both tolerance after the radiopharmaceutical administration and long-term toxicity.

## Simple Summary

This study investigates the safety of Radioligand Therapy with [^225^Ac]Ac-PSMA in patients with metastatic castration-resistant prostate cancer who have progressed after treatment with [^177^Lu]Lu-PSMA. The goal of this study was to retrospectively assess both Actinium-225-monotherapy and TANDEM therapy with [^225^Ac]Ac-/[^177^Lu]Lu-PSMA, focusing on tolerance after the radiopharmaceutical administration (89 patients), long-term safety and salivary gland function (71 patients), and survival (89 patients). The study examined hematological, renal, and hepatic toxicities, as well as the onset or worsening of xerostomia, demonstrating that [^225^Ac]Ac-PSMA therapy was generally well tolerated, with few severe adverse effects, primarily hematologic. These results emphasize the potential of [^225^Ac]Ac-PSMA as an effective therapy for advanced prostate cancer, especially regarding long-term tolerability, and may help guide future therapeutic decisions, optimize treatment protocols, and promote the further use of alpha-emitting therapies in oncology.

## 1. Introduction

Prostate cancer is one of the most common cancers in men [1], and it is the leading cause of cancer-related death in this population [2]. In the metastatic setting, patients who fail first-line hormone therapy develop castration-resistant tumors, which have a poor prognosis. These patients are typically treated with chemotherapy as second-line therapy; however, this presents a major clinical challenge [3,4].

In recent years, radiomolecular medicine has gained significant attention, particularly following the discovery of an excellent theranostic target: prostate-specific membrane antigen (PSMA). PSMA is a transmembrane enzyme (glutamate carboxypeptidase II) expressed on the surface of prostate tumor cells. It is upregulated in tumors with higher Gleason scores, during tumor de-differentiation, and in metastatic disease [5]. This makes PSMA an ideal candidate for molecular imaging due to its high sensitivity and specificity [6], as well as for radiopharmaceutical therapy. PSMA is also expressed in tumor neovasculature and in normal tissues such as the salivary glands, lacrimal glands, proximal renal tubules, and small intestine [5].

A significant milestone in the treatment of metastatic castration-resistant prostate cancer (mCRPC) was achieved with the VISION trial [7]. This trial demonstrated a marked improvement in overall survival and image-based progression-free survival in patients who had progressed after treatment with androgen receptor pathway inhibitors and taxane-based chemotherapy. Treatment with [^177^Lu]Lu-PSMA-617, combined with supportive care, significantly outperformed best supportive care alone [7]. Following these promising results, PSMA labeled with the beta-emitter Lutetium-177 was approved as [^177^Lu]Lu-Vipivotide Tetraxetan (Pluvicto^®^) by the U.S. Food and Drug Administration (FDA) in 2022 [8] and later by the European Medicines Agency (EMA) [9]. For patients who progress after [^177^Lu]Lu-PSMA Radioligand Therapy (PRLT), alpha-PRLT with [^225^Ac]Ac-PSMA offers a promising treatment option. In countries where Actinium-225 is available, such as Germany, alpha-PRLT with [^225^Ac]Ac-PSMA can be used either as monotherapy or in combination with [^177^Lu]Lu-labeled PSMA (TANDEM therapy) [10,11,12,13,14].

There are concerns about the uptake of radiolabeled PSMA by the salivary glands and its potential impact on gland function. This can lead to significant, irreversible damage, resulting in xerostomia (“sicca syndrome”) and a reduced quality of life. As a result, the salivary glands are considered dose-limiting organs during PRLT. The VISION trial reported that 38.8% of patients receiving [^177^Lu]Lu-PSMA-617 plus supportive care experienced dry mouth, compared to just 0.5% in the cohort with the best supportive care alone [7]. Nonetheless, none of these patients experienced severe xerostomia (G3). In another study, 26 patients with mCRPC received [^225^Ac]Ac-PSMA-617 PRLT after disease progression following [^177^Lu]Lu-PSMA. All patients experienced irreversible xerostomia (G1/G2), which began after the first cycle and worsened with additional cycles. Notably, 23% of these patients chose to discontinue treatment due to the severity of their xerostomia [14]. The exact mechanism behind PSMA uptake by salivary glands is not yet fully understood, and only limited data are currently available in the literature regarding lacrimal gland toxicity after PRLT.

We conducted a retrospective study to evaluate the safety of [^225^Ac]Ac-PSMA PRLT, both as monotherapy and in TANDEM with Lutetium-177. The focus was on adverse events after the radiopharmaceutical administration, long-term toxicity, and the impact on salivary gland function. We also assessed overall survival (OS) and follow-up duration.

## 2. Materials and Methods

We retrospectively enrolled 89 heavily pre-treated mCRPC patients who received at least one cycle of [^225^Ac]Ac-PSMA-I&T PRLT, either as monotherapy or in combination with [^177^Lu]Lu-PSMA-I&T (TANDEM therapy), after conventional therapeutic options had been exhausted. All patients were treated at CURANOSTICUM Wiesbaden-Frankfurt, Center for Advanced Radiomolecular Precision Oncology (Wiesbaden, Germany) under compassionate use between December 2020 and September 2024, in accordance with local radiation protection regulations. Due to the retrospective data acquisition and analysis of this investigation, ethical review and approval were waived by the institutional review board. All patients gave their written informed consent to receive therapy (PRLT). All procedures were performed in compliance with the German Medicinal Products Act, AMG §13 2b, the conditions of the Declaration of Helsinki article 37 “Unproven interventions in clinical practice”, and the responsible regulatory body (Government of Hesse).

The institutional eligibility criteria for PRLT have been published before [15]. In short, patients with mCRPC and prior taxane-based chemotherapy, ineligibility for chemotherapy, or explicit refusal of chemotherapy were suitable. A pretherapeutic [^68^Ga]Ga-PSMA-11 PET scan had confirmed intense PSMA expression of the tumor lesions. The dosing of Lutetium-177- and Actinium-225-labeled PSMA-I&T PRLT was patient-individualized based on, among other factors, the tumor burden, renal function, bone marrow reserve, and pre-treatments. The number of total cycles was never pre-determined but decided according to the patient response after each cycle. TANDEM PRLT with the combined administration of [^225^Ac]Ac-PSMA-I&T and [^177^Lu]Lu-PSMA-I&T was indicated in patients with a critically high tumor load documented at [^68^Ga]Ga-PSMA-11 PET or in patients who had failed or became refractory to [^177^Lu]Lu-PSMA-I&T monotherapy [16]. The clinical management and follow-up of PRLT were based on national and international consensus recommendations and guidelines, respectively [17,18]. A two-night hospitalization was required for each treatment.

The entire cohort (n = 89) was analyzed to assess the occurrence of adverse events during or immediately following [^225^Ac]Ac-PSMA administration, OS (calculated using the Kaplan–Meier method), and follow-up duration. Only patients with at least one follow-up (n = 71) were included in the analysis to evaluate the long-term safety. Despite our efforts, we were unable to collect follow-up data for 18 patients, as they were lost during follow-up due to various reasons, including patients coming from abroad preferring to continue treatment in their home country and patients unable to attend the following visit because of family or financial constraints. Notably, none of the patients discontinued treatment due to toxicity. Follow-up was carried out both as a post-treatment visit in a long-term care setting (all patients were seen and examined regularly 10 to 12 weeks after treatment for as long as they lived) and as visits at the next treatment cycle. The follow-up included both clinical evaluation and blood sample analyses. In this real-world setting, follow-up intervals were heterogeneous and varied among patients, with some intervals being shorter or longer than others.

Long-term safety was evaluated in terms of hematological, renal, and hepatic toxicity, as well as the onset or progression of xerostomia. All parameters were prospectively documented in a structured database and retrospectively analyzed. Both the tolerance after the radiopharmaceutical administration and long-term adverse events were graded according to the Common Terminology Criteria for Adverse Events (CTCAE) v.5.0, as proposed by the National Institutes of Health in 2017 [19]. Blood sample analysis included measurements of hemoglobin, white blood cell count, platelet count, creatinine, transaminases, alkaline phosphatase, and total bilirubin levels. To evaluate nephrotoxicity, we also assessed the estimated glomerular filtration rate, blood urea nitrogen, and electrolyte levels. In case of significant renal impairment, renal scintigraphy with [^99m^Tc]Tc-MAG3 was performed to assess tubular extraction function. However, these data are not reported as the main parameter, always measured, was the creatinine value. Salivary gland function was assessed using both unstimulated and stimulated objective methods. The unstimulated salivary flow was assessed using four dental rolls positioned in the gum cavities and left in place for five minutes. Stimulated salivary flow was evaluated by chewing a folded compress for two minutes. The weight difference between the dry and wet rolls was measured in grams, and the results from both assessments were summed and evaluated according to specific cutoffs [20,21]. Additionally, each patient completed a standardized questionnaire as described by Langbein et al. [15] to assess direct and indirect signs of xerostomia (e.g., dysphagia, salivary gland pain, stomatitis, dental pain, or caries) as well as eating habits to evaluate the impact on quality of life (e.g., use of water during chewing, lubricants, or purees/soft foods). Lacrimal gland function was also assessed through specific questions regarding dry eyes and conjunctivitis.

Patients received [^225^Ac]Ac-PSMA PRLT based on [^68^Ga]Ga-PSMA or [^18^F]F-PSMA PET/CT findings. Some patients underwent imaging modalities other than [^68^Ga]Ga-PSMA PET/CT to provide a better assessment of the disease (e.g., [^18^F]FDG PET/CT, CT, MRI), but these were not used as eligibility criteria for PRLT, nor were they included in the protocol for selection. All patients received intravenous administration of either ondansetron or granisetron prior to treatment, along with 4 mg of intravenous dexamethasone. Hydration and salivary gland protection were provided during each cycle. Salivary gland protection was performed in all patients using a combined approach: retroauricular scopolamine patches applied from three days before to two hours after the radiolabeled-PSMA injection [20,22], and chewing polyglutamate tablets following Paganelli’s protocol [23]. [^225^Ac]Ac-PSMA was administered via intravenous injection, with vital signs monitored during the process. In the case of TANDEM therapy, [^225^Ac]Ac-PSMA and [^177^Lu]Lu-PSMA were administered either on the same day or on consecutive days. Additionally, for TANDEM therapy, SPET/CT was performed the day after injection to assess the intensity and distribution of radiopharmaceutical uptake, including PSMA uptake by the salivary glands.

## 3. Results

### 3.1. Patient Cohort and PRLT Data

**Figure 1:**
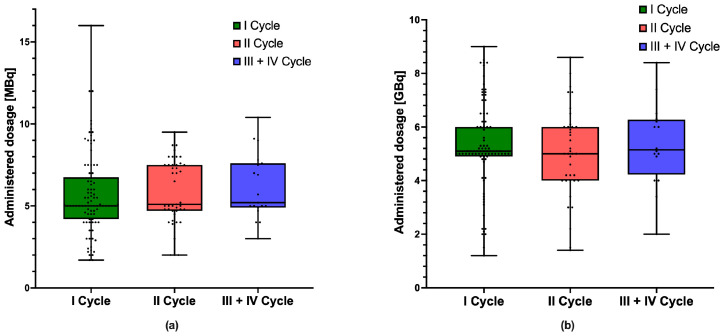
(**a**) Boxplots of the administered Actinium-225 dosages according to cycle, (**b**) Boxplots of the administered Lutetium-177 dosages according to cycle.

Our cohort included 89 heavily pre-treated mCRPC patients (age range 43–88 years at the time of their first [^225^Ac]Ac-PSMA PRLT). The majority of these patients (93.2%) were previously treated with androgen receptor pathway inhibitors, e.g., Abirateron or Enzalutamid; androgen deprivation therapy, such as Leuprorelin or Degarelix (87.6%); previously received radiotherapy for the primary tumor, lymph node metastases, or distant metastases (68.5%); surgery for the primary tumor, lymph node metastases, or distant metastases or interventional procedures, such as trans-arterial chemoembolization of liver metastases or electroporation of the primary tumor (60.7%); chemotherapy, such as Docetaxel or Cabazitaxel (51.7%); [^177^Lu]Lu-PSMA PRLT (92.1%); bone protection, such as Denosumab (58.4%); immuno-checkpoint inhibitors, such as Ipilimumab (20.2%); and PARP inhibitors, such as Olaparib (7.8%). The pattern of metastases included primarily bone (89% of patients), followed by lymph nodes (67%), liver (21%) and lung (2%); at the time of the first PRLT cycle, 58% of patients manifested both bone and lymph node metastases, and 17% of patients showed both bone and liver metastases. Over a period of 45 months, 151 [^225^Ac]Ac-PSMA cycles were administered (10 Actinium-225-monotherapies and 141 TANDEM PRLT using [^225^Ac]Ac-/[^177^Lu]Lu-PSMA). At the time of analysis (late September 2024), 46 patients had received one cycle, 28 patients had received two cycles, and 15 patients had received more than two cycles of therapy (11 patients received 3 cycles, 4 patients received 4 cycles). Figure 1 shows the boxplots for the dosages of Actinium-225 (a) and Lutetium-177 (b) administered in the first, second, and subsequent cycles, respectively.

### 3.2. Survival Analysis

**Figure 2:**
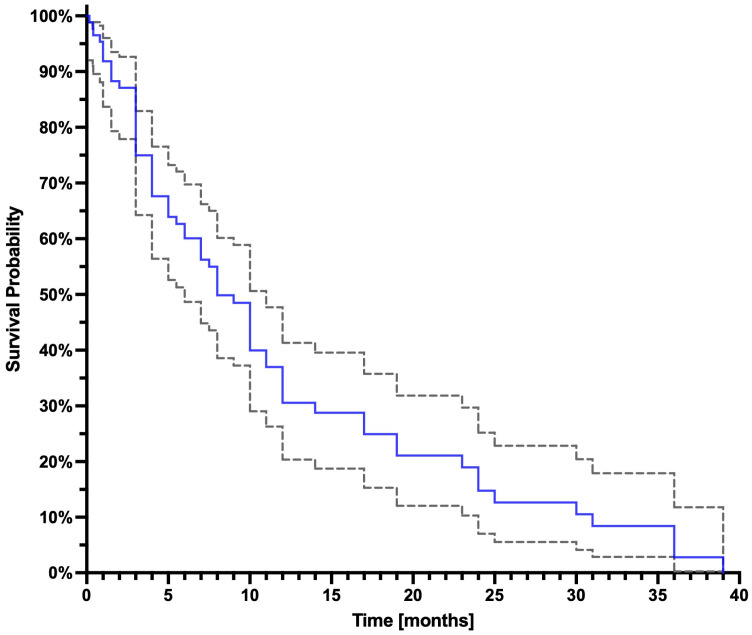
The blue line represents the Kaplan–Meier curve; the dashed gray lines represent the confidence interval 95%.

**Figure 3:**
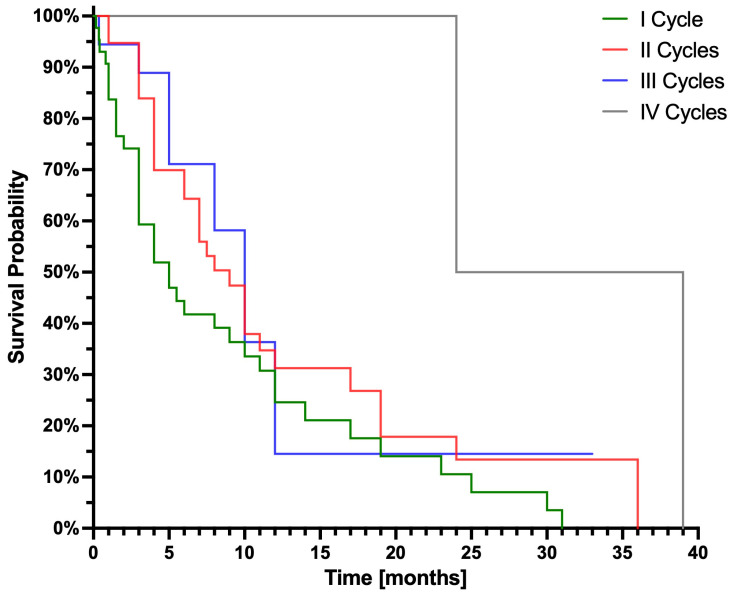
Kaplan–Meier curves according to the number of PRLT cycles administered as of late September 2024: I cycle, 46 patients; II cycles, 28 patients; III cycles, 11 patients; IV cycles, 4 patients.

At the time of analysis, 68 patients had passed away (survival ranging from 5 days to 39 months, median 7 months), and 21 patients were alive at evaluation (follow-up duration: 1 to 33 months; median: 9 months; interquartile range: 6.8 months). The Kaplan–Meier curve is presented in Figure 2, while Figure 3 illustrates Kaplan–Meier curves stratified by the number of PRLT cycles administered to the patients.

### 3.3. Tolerance After the Radiopharmaceutical Administration

The administration of PRLT was generally well tolerated, with only a few mild adverse events after the radiopharmaceutical administration (Grades 1 or 2 according to the CTCAE v5.0). The most common adverse event in this setting was flare pain (n = 16, 18%), primarily manifesting as bone pain in skeletal metastases following internal radiation exposure. Other adverse events related to the radiopharmaceutical administration included nausea (n = 8, 9%), emesis (n = 5, 5.6%), fever (n = 1, 1.1%), and headache (n = 1, 1.1%). All these adverse events were either self-limiting or, when necessary, easily managed with routine medications such as metamizole, ibuprofen, or antiemetics.

### 3.4. Hematological Toxicity

Hematological long-term toxicity was assessed in 71 patients considering hemoglobin levels, white blood cell count and platelet count. The management of severe long-term hematological toxicity was supportive therapy, including blood transfusions and administration of growth factors. The distribution of patients before and after treatment according to these parameters is displayed in Table 1.

**Figure 4:**
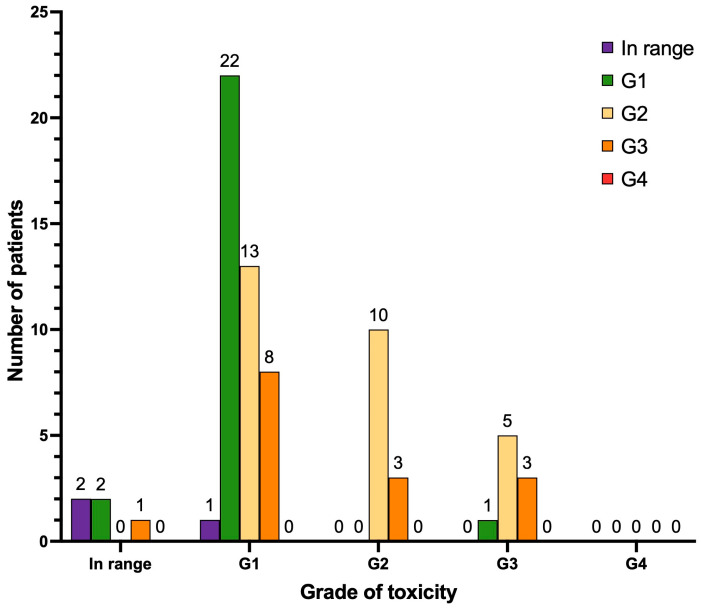
Graphical representation of hematological long-term toxicity regarding anemia (n = 71 patients). Legend (CTCAE v.5.0.): in range: hemoglobin (Hb) > lower limit of normal (LLN); anemia G1 (Hb < LLN–10 g/dL); anemia G2 (Hb 10–8 g/dL); anemia G3 (Hb < 8 g/dL), transfusion indicated; anemia G4 (life-threatening consequences), urgent intervention indicated.

With regard to anemia (Figure 4), after treatment, two patients manifested de novo anemia G1 (2.8%), and one patient manifested de novo anemia G3 (1.4%). Thirteen patients progressed from anemia G1 to G2 (18.3%), eight patients progressed from anemia G1 to G3 (11.3%) and three patients progressed from anemia G2 to G3 (4.2%). No cases of anemia G4 were observed. The remaining patients of the cohort either continued to have normal values of hemoglobin (n = 2) or the same grade of anemia as reported at baseline pre-PRLT (n = 22 anemia G1; n = 10 anemia G2; n = 3 anemia G3), or their hemoglobin levels improved during follow-up (n = 7).

**Figure 5:**
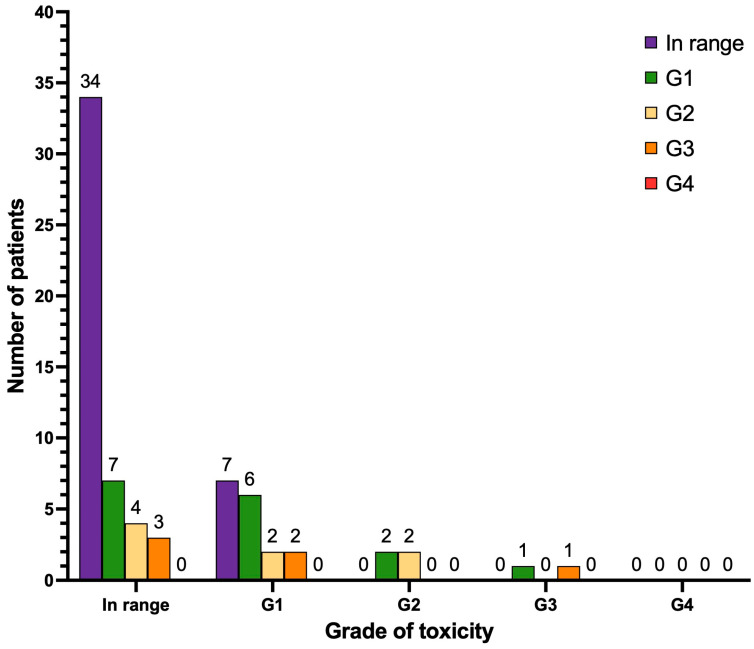
Graphical representation of hematological long-term toxicity regarding leukocytopenia (n = 71 patients). Legend (CTCAE v.5.0): in range, with blood cells (WBC) > lower limit of normal (LLN); leukocytopenia G1 (WBC < LLN–3000/mm^3^); leukocytopenia G2 (WBC 3000–2000/mm^3^); leukocytopenia G3 (WBC 2000–1000/mm^3^); leukocytopenia G4 (WBC < 1000/mm^3^).

When considering leukocytopenia (Figure 5), after treatment, seven patients manifested de novo leukocytopenia G1 (9.9%), four patients manifested de novo leukocytopenia G2 (5.6%), and three patients manifested de novo leukocytopenia G3 (4.2%). Two patients progressed from leukocytopenia G1 to G2 (2.8%) and two patients progressed from leukocytopenia G1 to G3 (2.8%). No cases of leukocytopenia G4 were observed. The remaining patients of the cohort either continued to have a normal white blood cell count (n = 34) or the same grade of leukocytopenia as reported at baseline pre-PRLT (n = 6 leukocytopenia G1; n = 2 leukocytopenia G2; n = 1 leukocytopenia G3), or their white blood cell count improved during follow-up (n = 10).

**Figure 6:**
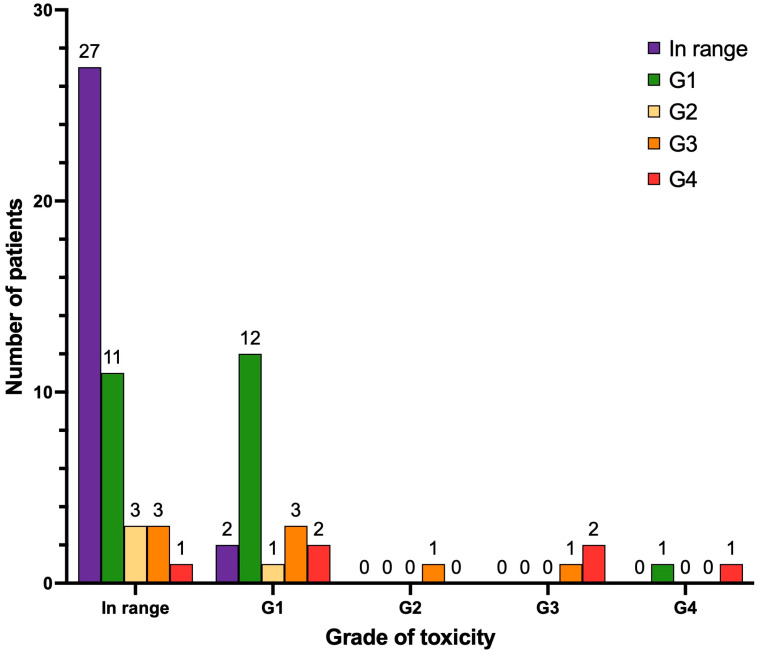
Graphical representation of hematological long-term toxicity regarding thrombocytopenia (n = 71 patients). Legend (CTCAE v.5.0): in range: platelets (PTL) > lower limit of normal (LLN); thrombocytopenia G1 (PTL < LLN–75.000/mm^3^); thrombocytopenia G2 (PTL 74.000–50.000/mm^3^); thrombocytopenia G3 (PTL 49.000–25.000/mm^3^); thrombocytopenia G4 (PTL < 25.000/mm^3^).

With regard to thrombocytopenia (Figure 6), after treatment, 11 patients developed de novo thrombocytopenia G1 (15.5%), 3 patients developed de novo thrombocytopenia G2 (4.2%), 3 patients developed de novo thrombocytopenia G3 (4.2%), and 1 patient developed de novo thrombocytopenia G4 (1.4%). One patient progressed from thrombocytopenia G1 to G2 (1.4%), three patients progressed from thrombocytopenia G1 to G3 (4.2%), two patients progressed from thrombocytopenia G1 to G4 (2.8%), one patient progressed from thrombocytopenia G2 to G3 (1.4%) and two patients progressed from thrombocytopenia G3 to G4 (2.8%). The remaining patients of the cohort either continued to have normal platelet count (n = 27) or the same grade of thrombocytopenia as reported at baseline pre-PRLT (n = 12 thrombocytopenia G1; n = 1 thrombocytopenia G3; n = 1 thrombocytopenia G4), or their platelet count improved during follow-up (n = 3).

### 3.5. Nephrotoxicity and Hepatotoxicity

Long-term nephrotoxicity and hepatotoxicity were assessed in 71 patients considering levels of creatinine, transaminases, alkaline phosphatase and total bilirubin. The distribution of patients before and after treatment according to these parameters is displayed in Table 2.

**Figure 7:**
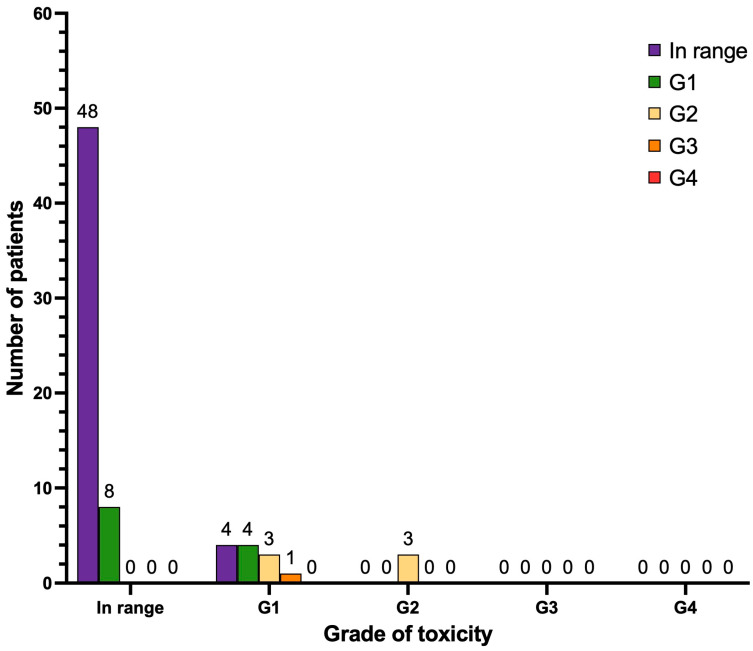
Graphical representation of long-term nephrotoxicity (n = 71 patients). Legend (CTCAE v.5.0): in range, serum creatinine < upper limit of normal (ULN); renal insufficiency G1 (serum creatinine 1.0–1.5 × ULN); renal insufficiency G2 (1.5–3.0 × ULN); renal insufficiency G3 (3.0–6.0 × ULN); renal insufficiency G4 (> 6.0 × ULN).

With regard to nephrotoxicity (Figure 7), after treatment, 48 patients did not manifest any degree of renal impairment (67.6%) and 8 patients developed de novo renal impairment G1 (11.3%). Three patients progressed from renal impairment G1 to G2 (4.2%), and one patient progressed from renal impairment G1 to G3 (1.4%). No cases of renal impairment G4 were observed. Other patients of our cohort either continued to have the same degree of renal impairment as documented at baseline pre-PRLT (n = 4 renal impairment G1; n = 3 renal impairment G2) or their creatinine levels improved during follow-up (n = 4).

**Figure 8:**
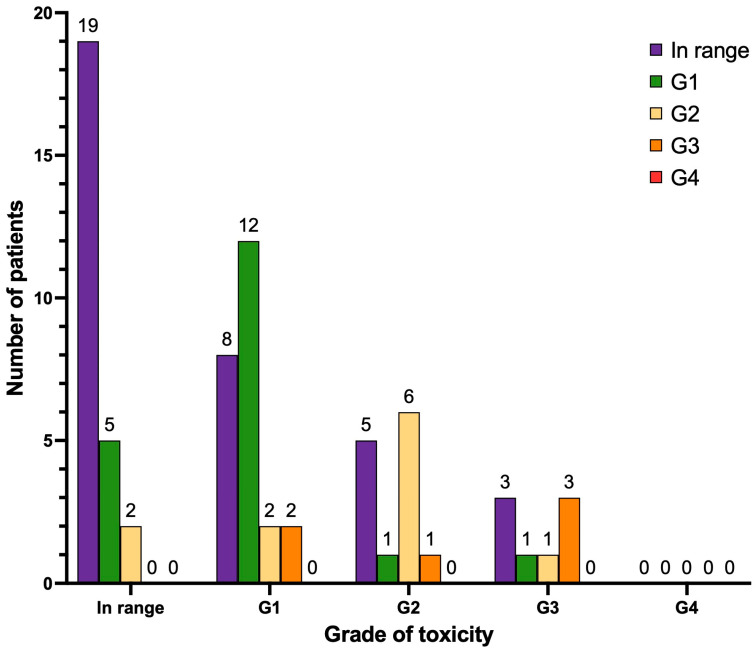
Graphical representation of long-term hepatotoxicity (n = 71 patients). Legend (CTCAE v.5.0): in range, aspartate aminotransferase (AST) and alanine aminotransferase (ALT) < upper limit of normal (ULN); hypertransaminasemia G1: > ULN–3.0 × ULN; G2: 3.0–5.0 × ULN; G3: 5.0–20.0 × ULN; G4: > 20.0 × ULN. In range, alkaline phosphatase (ALP) < ULN; G1: > ULN–2.5 × ULN; G2: 2.5–5.0 × ULN; G3: 5.0–20.0 × ULN; G4: > 20.0 × ULN. In range, total bilirubin < ULN; G1: > ULN–1.5 × ULN; G2: 1.5–3.0 × ULN; G3: 3.0–10.0 × ULN; G4: > 10.0 × ULN.

With regard to hepatotoxicity (Figure 8), after treatment, 19 patients did not manifest any degree of liver toxicity (26.8%), 5 patients developed de novo liver impairment G1 (7%), and 2 patients developed de novo liver impairment G2 (2.8%). Two patients progressed from liver impairment G1 to G2 (2.8%), two patients progressed from liver impairment G1 to G3 (2.8%), and one patient progressed from liver impairment G2 to G3 (1.4%; this patient presented with hepatic metastases at [^68^Ga]Ga-PSMA PET/CT). No cases of liver impairment G4 were observed. Other patients of our cohort either continued to have the same degree of liver impairment as documented at baseline pre-PRLT (n = 12 liver impairment G1; n = 6 liver impairment G2; n = 3 liver impairment G3) or their hepatic function improved during follow-up (n = 19).

### 3.6. Xerostomia (Dry Mouth)

The onset or worsening of xerostomia (“sicca syndrome”) was assessed in 71 patients according to the scores of stimulated and unstimulated methods of salivary glands’ functional assessment and the results of a standardized questionnaire [15]. The distribution of patients before and after treatment according to this symptom is displayed in Table 3.

**Figure 9:**
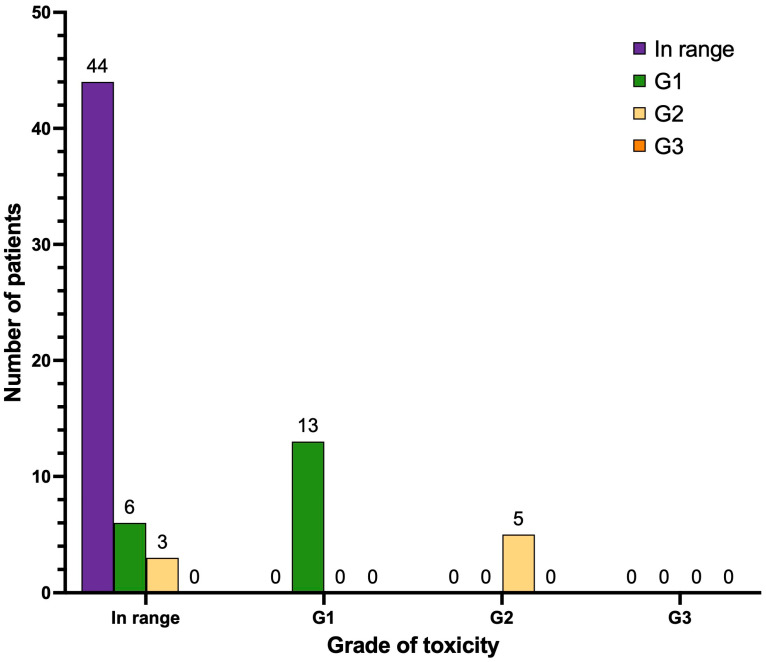
Graphical representation of the onset or progression of xerostomia (n = 71 patients). Legend (CTCAE v.5.0): in range, no signs or symptoms of dry mouth; xerostomia G1: symptomatic (e.g., dry or thick saliva) without significant dietary alteration, unstimulated saliva flow > 0.2 mL/min; xerostomia G2: moderate symptoms, oral intake alterations (e.g., copious water, lubricants, diet limited to purees-soft food), unstimulated saliva flow 0.1–0.2 mL/min; xerostomia G3: inability to adequately aliment orally, tube feeding or total parental nutrition indicated, unstimulated saliva flow < 0.1 mL/min.

After treatment (Figure 9), the majority of patients in our cohort did not manifest any degree of dry mouth (n = 44). Six patients manifested de novo xerostomia G1 (8.4%), and three patients manifested de novo xerostomia G2 (4.2%). No cases of xerostomia G3 were observed. None of the patients with pre-PRLT xerostomia (n = 18, distributed as follows: n = 13 with xerostomia G1 and n = 5 with xerostomia G2) showed a progression of the degree of symptoms after therapy. Most importantly, none of the patients of our cohort had to discontinue the treatment due to xerostomia.

Regarding lacrimal gland toxicity, patients were specifically asked if they were experiencing symptoms of dry eyes, like stinging and burning sensations in the eyes, blurred vision, a scratchy or gritty feeling, redness or irritation, or sensitivity to light. However, none of these symptoms were reported by patients, and if present prior to treatment, they did not worsen following [^225^Ac]Ac-/[^177^Lu]Lu-PSMA-I&T PRLT.

## 4. Discussion

Patients suffering from chemotherapy-resistant mCRPC represent one of the most significant therapeutic challenges in oncology. PRLT has the potential to improve therapeutic management by targeting PSMA. PRLT using the beta-emitter Lutetium-177 has already been demonstrated to be safe, with rare adverse effects after the radiopharmaceutical administration and a few reported cases of long-term severe hematological, renal, or hepatic toxicity as well as severe cases of xerostomia [7]. It has proven effective for pain relief, radiological response and survival outcomes. More recently, PSMA ligand labeled with the alpha-emitter Actinium-225 has been proposed in patients progressing after PRLT with beta-emitters, especially to treat smaller metastatic lesions and more disseminated patterns of disease, due to the higher linear energy transfer and shorter tissue penetration range of alpha particles compared to beta particles [24].

The results of our retrospective analysis with prospective documentation indicate that [^225^Ac]Ac-PSMA PRLT in heavily pre-treated mCRPC patients is overall safe, with only a few transient and mild adverse events after the radiopharmaceutical administration (self-limiting or manageable with routine medications). Severe long-term toxicities (G3/G4) were rare and included hematological, renal, and hepatic adverse events. Nonetheless, in patients receiving TANDEM PRLT, it was impossible to differentiate toxicity attributable to Actinium-225 from toxicity attributable to Lutetium-177. Moreover, the reported long-term toxicities might also be caused by other factors, e.g., previous chemotherapy, disease progression with bone marrow replacement, and liver involvement in therapy-refractory cases. When considering xerostomia in patients receiving [^225^Ac]Ac-PSMA PRLT with appropriate salivary glands’ protection, this therapy only minimally influenced the onset of xerostomia (only nine new cases, all G1/G2) and did not influence the progression of pre-existing xerostomia. In fact, the incidence or progression of pre-existing xerostomia can be effectively reduced by appropriate methods for salivary glands’ protection (e.g., scopolamine patches, chewing of polyglutamate tablets, or intra-parotid botulinum injection). Moreover, none of the patients developed severe xerostomia or had to discontinue PRLT due to xerostomia. Kratochwil et al. reported about the first-in-human administration of [^225^Ac]Ac-PSMA-617 as salvage therapy in two patients with clinically critical mCRPC, who had progressed under conventional therapies [10], benefiting from alpha-PRLT without relevant hematologic toxicity. However, both of them manifested xerostomia (of moderate grade in one patient; of severe grade, requiring spray substitution of saliva, in the other patient). Feuerecker et al. retrospectively studied a larger cohort of mCRPC patients (twenty-six) previously treated with standard therapies, who received [^225^Ac]Ac-PSMA-617 PRLT under compassionate use [14]. In this study, the authors reported a few cases of hematological G3/G4 toxicities—anemia (35%), leukocytopenia (27%), and thrombocytopenia (19%)—with two patients who stopped alpha-PRLT due to hematological toxicity. Moreover, all patients experienced xerostomia G1/G2, with six of them who discontinued the treatment due to this side effect, unlike in our experience. A recent review summarizing the clinical data of both [^177^Lu]Lu-PSMA and [^225^Ac]Ac-PSMA PRLT [25] pointed out that the most common toxicity in patients treated with [^225^Ac]Ac-PSMA is xerostomia, overall higher than that occurring after [^177^Lu]Lu-PSMA (xerostomia of any grade: 77% vs. 37%), with 17% of G3 or higher grade of dry mouth. Furthermore, the frequency and severity of hematological toxicities were quite comparable between [^225^Ac]Ac-PSMA and [^177^Lu]Lu-PSMA PRLT: anemia G3 or higher 11% vs. 10%; leukocytopenia G3 or higher 9% vs. 2%; thrombocytopenia G3 or higher 7% vs. 7%. Interestingly, Sathekge et al. retrospectively studied the survival and toxicity of [^225^Ac]Ac-PSMA-617 PRLT in 21 patients with de novo metastatic hormone-sensitive prostate carcinoma (mHSPC), who had refused standard treatment options (e.g., androgen deprivation therapy and chemotherapy) [26]. The administration of [^225^Ac]Ac-PSMA-617 was overall well tolerated, with xerostomia G1/G2 being the most common adverse event (94% of patients), with no cases of dry eyes and no significant change in platelet or white blood cell count between pre- and post-treatment values; only ten patients manifested a significant decrease in hemoglobin levels after treatment. In addition, this study showed good survival results in the entire population of mHSPC patients (median OS 31 months).

The precise mechanism behind PSMA uptake by the salivary glands is not yet fully understood. Despite the high PSMA uptake observed in PET/CT scans, this does not correlate with the relatively low physiological expression of PSMA in salivary gland tissue. Several hypotheses have been proposed to explain this discrepancy. Some researchers investigated the salivary gland uptake of [^177^Lu]Lu-PSMA-617 using autoradiography and analyzed both specific and non-specific uptake in pig salivary glands. They found that a significant portion of the uptake was non-specific [27]. Other studies found that the high accumulation of PSMA radioligands in salivary glands observed with [^68^Ga]Ga-PSMA-11 PET did not correlate with high PSMA expression levels in autoradiography and immunohistochemistry. This suggests that the marked accumulation of PSMA radioligands in salivary glands is not primarily driven by specific PSMA-mediated uptake [28].

Several methods have been employed in recent years to reduce PSMA uptake by the salivary glands. These include applying ice packs to the parotid glands for local cooling, although the impact of icepacks on PSMA uptake seems to be limited [29], or using lemon juice to stimulate salivation [30]. Baum et al. demonstrated a reduction in the SUVmean of up to 64% in the right parotid gland compared to baseline in a [^68^Ga]Ga-PSMA PET/CT scan performed 45 days after intraparenchymal injections of botulinum toxin A in a 63-year-old patient. This reduction was attributed to the suppression of salivary gland metabolism [31]. This approach was studied more systematically by Müller et al. using high-dose botulinum for salivary gland protection [20]. Paganelli et al. proposed a protocol to reduce salivary gland uptake in mCRPC patients undergoing PRLT with [^177^Lu]Lu-PSMA-617, which was confirmed by dosimetry [23]. This protocol involves the oral administration of polyglutamate folate tablets of plant origin as parotid gland protectors, according to the following regimen: two tablets half an hour before treatment, two during treatment, and two tablets four hours after treatment. These tablets contain glutamate groups that act as substrates for PSMA enzymatic activity, stimulating the proteolytic activity of PSMA in the salivary glands. Further studies are needed to better understand the mechanisms behind PSMA uptake in salivary glands. This knowledge could help mitigate salivary gland toxicity after PRLT, potentially allowing for the administration of higher doses and enabling dose adjustments based on the patient’s individual characteristics.

PSMA uptake is also observed in the lacrimal glands on both [^68^Ga]Ga-PSMA-PET/CT and [^177^Lu]Lu-PSMA SPET/CT, and dry eye syndrome may occur after alpha-PRLT. Pepin et al. reported a patient experiencing dry eye G3 with concomitant blepharitis after [^177^Lu]Lu-PSMA-617 PRLT; this condition was treated with a neomycin–polymyxin–dexamethasone ophthalmic suspension, artificial tears and topical medications, and oral antihistamines [32]. However, no lacrimal gland toxicity was noted in our patient cohort, and no symptoms were reported by patients. If symptoms were present before treatment, they did not worsen following the alpha-PRLT or TANDEM PRLT.

Furthermore, this study demonstrated promising survival outcomes with [^225^Ac]Ac-PSMA PRLT, with survival reaching up to 39 months in our cohort. Nonetheless, this study is not a prospective randomized clinical trial and contains some heterogenous data; therefore, this result has to be considered anecdotal and needs confirmation from standardized clinical studies.

[^225^Ac]Ac-PSMA PRLT can be administered as monotherapy or in TANDEM with [^177^Lu]Lu-PSMA, exploiting the physical properties of both nuclides [15,33,34,35]. This opens the possibility of using TANDEM PRLT either as initial therapy or as salvage therapy in mCRPC patients progressing after beta-PRLT. Additionally, in tumors resistant to internal radiation, PRLT can be administered concurrently with other therapies to enhance therapeutic efficacy (COMBO-PRLT) [36], such as external beam radiation therapy for localized lesions, immunotherapy, immune checkpoint inhibitors, DNA-damage response inhibitors in patients with BRCA1/2 mutations [37,38], and radiosensitizing agents [39]. For patients presenting with one or a limited number of PSMA-negative or weakly positive lesions, PRLT remains a viable option, particularly when combined with external beam radiation therapy. This approach can be effective in patients undergoing [^18^F]FDG PET/CT in addition to [⁶⁸Ga]Ga-PSMA PET/CT, especially in cases with FDG-positive but PSMA-negative lesions [40] or lytic bone metastases, with the aim to stabilize the bone and prevent pathological fractures. Recent studies and clinical applications have also explored labeling PSMA with Lead-212, a beta-emitter with alpha-emitting daughters [41].

## 5. Conclusions

PSMA labeled with the alpha-emitter Actinium-225 ([^225^Ac]Ac-PSMA) represents a promising therapeutic option for patients with advanced mCRPC who have progressed after PRLT using [^177^Lu]Lu-PSMA. However, adequate protection is essential to prevent the impairment of the salivary glands. Alpha-PRLT with [^225^Ac]Ac-PSMA (either as monotherapy or in TANDEM) in mCRPC patients who have failed previous treatments, including beta-PRLT and chemotherapy, is overall safe with promising survival outcomes, representing a flourishing clinical and research field in radiotheranostics. Prospective, multicentric randomized clinical trials are needed to confirm our single-institution experience.

## Figures and Tables

**Table 1:** Distribution of patients before and after PRLT considering anemia, leukocytopenia, and thrombocytopenia.

	Before Treatment		After Treatment	
**Anemia (Grading)**	Number (n)	Percent (%)	Number (n)	Percent (%)
In range	5	7.0	3	4.2
G1	44	62.0	25	35.2
G2	13	18.3	28	39.4
G3	9	12.7	15	21.1
G4	0	0	0	0
**Leukocytopenia (Grading)**	Number (n)	Percent (%)	Number (n)	Percent (%)
In range	48	67.6	41	57.7
G1	17	23.9	16	22.5
G2	4	5.6	8	11.3
G3	2	2.8	6	8.4
G4	0	0	0	0
**Thrombocytopenia (Grading)**	Number (n)	Percent (%)	Number (n)	Percent (%)
In range	45	63.4	29	40.8
G1	20	28.2	24	33.8
G2	1	1.4	4	5.6
G3	3	4.2	8	11.3
G4	2	2.8	6	8.4

**Table 2:** Distribution of patients before and after PRLT considering renal and hepatic function.

	Before Treatment		After Treatment	
**Renal function (Grading)**	Number (n)	Percent (%)	Number (n)	Percent (%)
In range	56	78.9	52	73.2
G1	12	16.9	12	16.9
G2	3	4.2	6	8.4
G3	0	0	1	1.4
G4	0	0	0	0
**Hepatic function (Grading)**	Number (n)	Percent (%)	Number (n)	Percent (%)
In range	26	36.6	35	49.3
G1	24	33.8	19	26.8
G2	13	18.3	11	15.5
G3	8	11.3	6	8.4
G4	0	0	0	0

**Table 3:** Distribution of patients before and after PRLT considering xerostomia.

	Before Treatment		After Treatment	
**Xerostomia** **(Grading)**	Number (n)	Percent (%)	Number (n)	Percent (%)
In range	53	74.6	44	62.0
G1	13	18.3	19	26.8
G2	5	7.0	8	11.3
G3	0	0	0	0

## Data Availability

The raw data supporting the conclusions of this article will be made available by the authors upon request.
